# Genotypic and tissue-specific variation of *Populus nigra* transcriptome profiles in response to drought

**DOI:** 10.1038/s41597-022-01417-z

**Published:** 2022-06-14

**Authors:** Christian Eckert, Henning Wildhagen, Maria João Paulo, Simone Scalabrin, Johannes Ballauff, Sabine K. Schnabel, Vera Vendramin, Joost J. B. Keurentjes, Marie-Béatrice Bogeat-Triboulot, Gail Taylor, Andrea Polle

**Affiliations:** 1 Forest Botany and Tree Physiology, University of Goettingen, Büsgenweg 2, Göttingen, Germany; 2 HAWK University of Applied Sciences and Arts, Faculty of Resource Management, Büsgenweg 1a, 37077 Göttingen, Germany; 3 Biometris, Wageningen UR Wageningen Plant Research, Droevendaalsesteeg 1, Wageningen, The Netherlands; 4 IGA Technology Services, via Jacopo Linussio 51, Udine, Italy; 5 Laboratory of Genetics, Wageningen University & Research, Droevendaalsesteeg 1, Wageningen, The Netherlands; 6 Université de Lorraine, AgroParisTech, INRAE, UMR Silva, Nancy, France; 7 Department of Plant Sciences, University of California, One Shields Ave, Davis, CA USA

**Keywords:** Drought, Abiotic

## Abstract

Climate change is one of the most important challenges for mankind in the far and near future. In this regard, sustainable production of woody crops on marginal land with low water availability is a major challenge to tackle. This dataset is part of an experiment, in which we exposed three genetically differentiated genotypes of *Populus nigra* originating from contrasting natural habitats to gradually increasing moderate drought. RNA sequencing was performed on fine roots, developing xylem and leaves of those three genotypes under control and moderate drought conditions in order to get a comprehensive dataset on the transcriptional changes at the whole plant level under water limiting conditions. This dataset has already provided insight in the transcriptional control of saccharification potential of the three *Populus* genotypes under drought conditions and we suggest that our data will be valuable for further in-depth analysis regarding candidate gene identification or, on a bigger scale, for meta-transcriptome analysis.

## Abstract (summary)

**Table:** 

Measurement(s)	transcriptome
Technology Type(s)	illumina sequencing
Factor Type(s)	treatment
Sample Characteristic - Organism	Populus nigra
Sample Characteristic - Environment	greenhouse experiment

## Background & Summary

Ongoing climate change, entailing more frequent and severe drought events, put biomass productivity at an increasing risk^1–3^, especially on marginal land. Considering the increasing demand for both, food production and feedstock, energy crop production systems should preferentially utilize perennial crops grown on marginal sites^4^. Consequently, there is a need to develop new germplasms of perennial biomass crops characterized by high productivity and the ability to maintain productivity under water limited growth conditions^5^. To this end, it is pivotal to understand the underlying physiological mechanisms and molecular drivers of drought stress responses and growth adjustment of trees, especially mechanisms underlying adjustments of growth in response to water deprivation.

Because of its adaptation to a broad range of habitats, its fast growth and available molecular and genomic resources, the genus *Populus* is considered as a model system for woody biomass plants^6^. The publication of the full genome sequence of *Populus trichocarpa*^7^ fostered the application of systems biology approaches on a multitude of *Populus* species, including most widespread species like *P. tremuloides* and its European counterpart *P. tremula*. In Europe, *P. nigra*, European black poplar, is also widely distributed, and this is reflected in significant phenotypic variation in growth rates, tree architecture and leaf size^8^*. P. nigra* is a candidate for second generation biofuels that use lignocellulosic biomass^9–11^ as well as a raw material for pulp and paper production^12^. As a pioneer species, *P. nigra* can also be used to preserve local biodiversity, as it is able to outcompete exotic poplar species. Therefore, it is used in restoration and protection of riparian forest sites^13^. In Eastern Europe, *P. nigra* is planted for soil protection and used for restoration areas polluted by industrial usage^14^. As a species perceived as both, economically and ecologically important, *P. nigra* is targeted in studies aiming to elucidate the genetic basis of variation in adaptive traits^8,15,16^.

In order to characterize traits that play a conserved role across populations adapted to contrasting natural habitats we included three *P. nigra* genotypes originating from areas with different water availability. The genotypes were previously shown to be genetically differentiated^8^. This genetic variance between the three subgroups originates from the last glacial period, where there were three refugia for *P. nigra*^8^ in Europe from which the species recolonized Europe. Among the three genotypes, the Spanish genotype grows on the driest land, while the Italian genotype derives from the most humid area^8,17^.

Here, trees of each genotype were exposed to a gradually increasing, precisely controlled, moderate drought for five weeks before harvest. Transcriptome profiles of the developing xylem, fine roots, and fully-expanded young leaves were prepared in order to get a holistic insight into the transcriptional reprogramming in specific tissues and between different genotypes. Of these tissues roots sense drying soil first^18,19^; the xylem is the water conducting system in plants^20^; and leaves play the key role in transpiration and gas exchange^21^. Thus, this study covers all pivotal tissues involved in the water balance of plants.

Our data set opens the possibility for analyses in various directions. One could either look at conserved gene clusters across all three genotypes to identify gene families that are important for drought acclimation or other important traits. An example for this kind is the investigation of the transcriptional drought response in the developing xylem^22^, which constitutes a subset of the present data. Weighted gene correlation network analysis identified genes correlated significantly with the saccharification potential of the wood, which was enhanced under drought^22^. Interestingly, no genes involved in lignin biosynthesis were found to be correlated with drought, but polysaccharide biosynthesis genes were upregulated, underpinning the improved saccharification potential^22^. This study gave a first glimpse in the power of the data set generated in this study. In-depth analyses of the whole data set regarding the acclimation of specific tissues to drier conditions and the crosstalk between tissues has not been done yet. In addition, investigations of the intraspecies genetic variation can assist identifying genetic markers that can be used for predictive breeding. Previous studies have mostly concentrated on intraspecific variation in single tissues^23,24^. Our dataset combines data on different tissues and genotypes. We therefore believe that this data set will be of keen interest for the scientific community and can add a piece to the puzzle to understand drought adaptation of trees.

## Methods

### Plant material

Three genotypes of *Populus nigra* L., originating from natural populations in France, Italy and Spain were studied. The three genotypes represent clones of individual trees sampled from the populations Drôme 6 (FR-6), La Zelata (IT1), and Ebro 2 (SP-2)^8^. Cuttings were planted in 10-liter plastic pots filled with a 1:1 (v/v) mixture of peat and sand fertilized with a slow-release fertilizer (4 g L^−1^ of Nutricote T100, 13:13:13 NPK and micronutrients; FERTIL S.A.S, Boulogne Billancourt, France). Plants were grown in two chambers of a glasshouse located at Champenoux, France (48°45′09.3″N, 6°20′27.6″E), under natural light conditions. Growth conditions in the greenhouse were affected by weather conditions, but the temperature was adjusted to not exceed 28 °C, and daily maxima of irradiance ranged from 73–478 W m^−2^. Plants were watered three times per day to field capacity on a custom-made automated watering system.

### Drought experiment

**Fig. 1:**
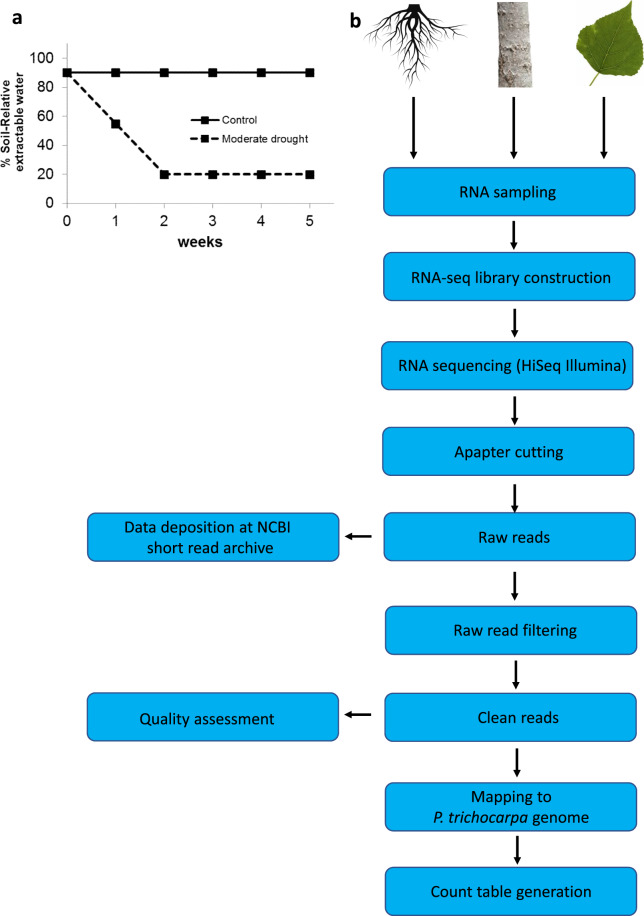
Experimental setup and data processing pipeline. (**a**) Water availability was reduced for two weeks to reach 20% soil extractable water (SEW) and was kept constant for 3 more weeks until harvest. (**b**) Flow chart of RNA sample processing from harvest to data analysis.

After six weeks of growth, plants of each genotype were randomly assigned to either a control or a drought treatment. The plants were allocated to the two greenhouse chambers in balanced proportions according to genotype and treatment. Plants were exposed to drought by gradually decreasing the available soil water content. The regulation of soil water availability was based on the soil relative extractable water content (REWsoil), which is defined as: REWsoil = (SWC - water content at wilting point)/(water content at field capacity - water content at wilting point), with SWC: soil volumetric water content; water content at wilting point = 3%; water content at field capacity = 32%. Control plants were watered to 85% REWsoil three times per day for the whole 5-week period of the experiment. For drought-treated plants, a target level of 20% REWsoil was defined, which was reached two weeks after starting to gradually withhold water. Plants were watered at this target level of REWsoil for the following three weeks^22,25^ (Fig. 1a).

After five weeks of control or drought treatment, all plants were harvested destructively. We used four individual plants from each genotype and water level (3 genotypes × 4 plants × 2 water levels = 24 plants) for the harvest of 3 tissues ( = 72 samples for further analysis). Different tissues per plant were harvested in parallel by several persons to achieve rapid sampling times. Leaf number 10 from the top of each plant was cut off, weighed and flash frozen in liquid nitrogen. The stem was cut at the base, the bark was removed from the lower part and the developing xylem, a soft tissue, was scratched from the surface and immediately frozen in liquid nitrogen. The root system with soil was carefully removed from the pot and briefly washed under tap water to clean fine roots, which were then cut off. The surface water from the collected fine roots was quickly dabbed off by tissue paper and then roots were frozen in liquid nitrogen. The samples were stored at −80 °C. For the extraction of RNA, each tissue was milled to a fine powder keeping the sample frozen by cooling the device under liquid nitrogen. Of each sample 150 mg of frozen tissue was weighed into the extraction medium for RNA. The procedure of RNA sampling to count table is depicted in Fig. 1b.

### RNA extraction, library preparation and sequencing

Total RNA was extracted from homogenized samples of a young fully expanded leaf, fine roots, and developing xylem of four biological replicates per genotype and treatment using the CTAB protocol^26^ with minor modifications described in^27^. After checking quality and integrity (see Technical Validation), 2 µg of total RNA were used for library preparation using the ‘TruSeq mRNA Sample Prep kit v2’ (Illumina, San Diego, CA, USA), following the manufacturer’s instructions. Libraries were then processed with Illumina cBot for cluster generation on the flowcell, following the manufacturer’s instructions and sequenced in 50 bp single-end mode at the 6-fold multiplex on the Illumina HiSeq2000 (Illumina, San Diego, CA, USA).

## Data Records

Raw data of RNA-seq analysis are deposited at the NCBI short read archive under SRP numbers SRP095832^28^ (dev. xylem samples) and SRP101711^29^ (fine root and leaf samples). Each biological replicate refers to a single SRA Sample Accession (SRS accession, Online-only Table 1). Raw count data are available at figshare data repository under 10.6084/m9.figshare.17031842.v3^30^. Tables for differentially expressed genes for each tissue and genotype are available under 10.6084/m9.figshare.19382603.v1^31^.

## Technical Validation

RNA integrity was determined using Agilent 2100 Bioanalyzer RNA Nano assay (Agilent technologies, Santa Clara, CA, USA). Average RIN values were 7.9 ± 0.5 for the developing xylem, 7.2 ± 0.4 for fine roots and 7.2 ± 0.3 for leaf samples. qRT-PCR validation of selected genes has been performed by Wildhagen and colleagues^22^.

### Quality assessment

**Fig. 2:**
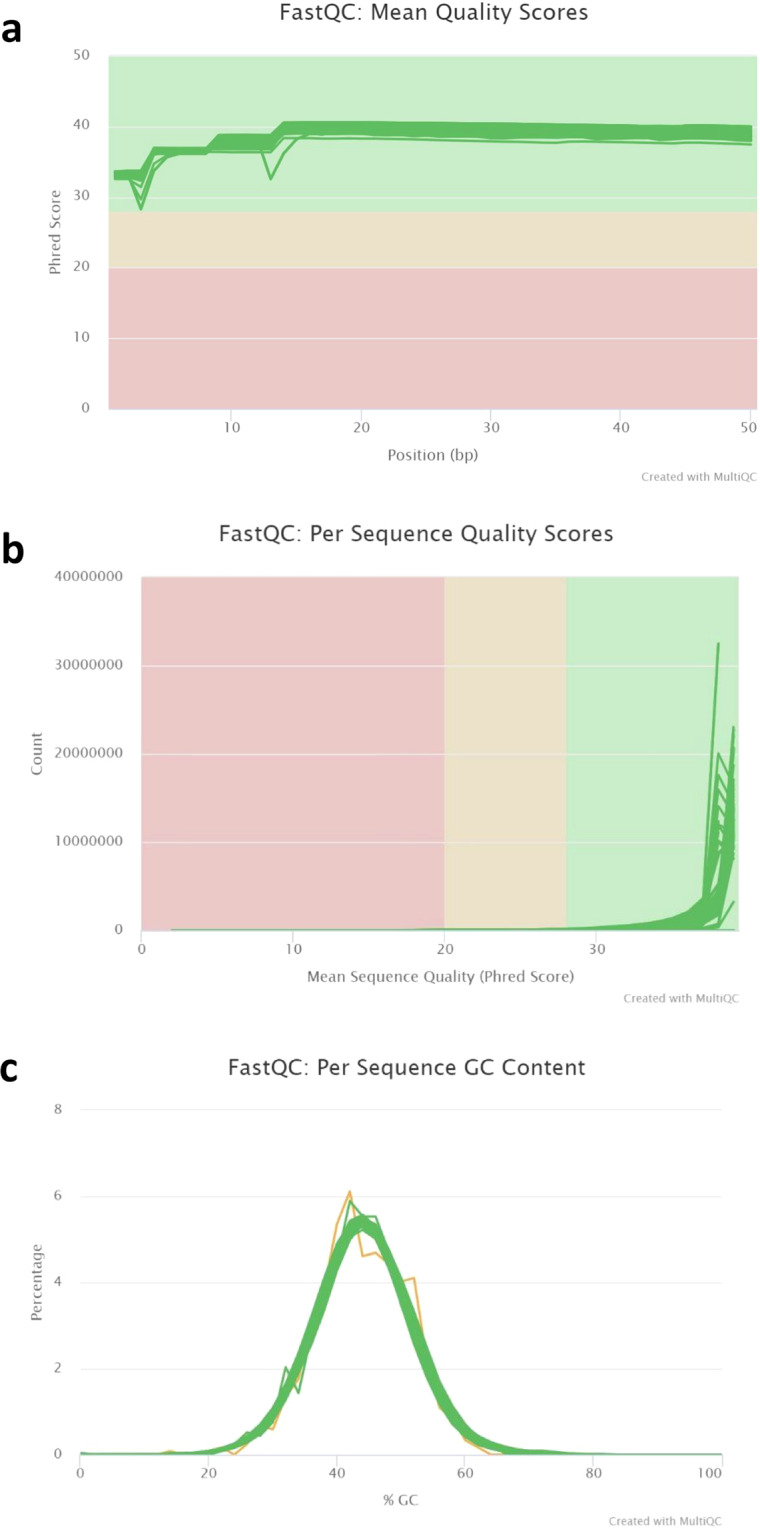
Quality assessment of RNA sequencing data. (**a**) Mean quality phred scores of individual samples, (**b**) Per Sequence Quality Scores as counts per phred score (**c**) GC content of individual samples. FastQC results for single samples were summarized using MulitQC.

FastQC Version 0.11.9 was applied to the clean reads to assess the quality of the sequence reads^32^. A summary of FastQC reports was generated using MultiQC^33^. Figure 2 shows that the Mean Quality Scores and the Per Sequence Quality Scores of all sequencing results were of high quality indicated by the green color in the diagrams. Per Sequence GC content was consistent at 44–45%.

### Data filtering and processing

Raw sequence reads were processed with the Python package Cutadapt v1.4.2^34^ to remove residual adapter contamination. Reads were subsequently trimmed to remove low-quality reads (option -trim_qual_left/right = 25) and reads shorter than 40 nucleotides, using the PRINSEQ software v.lite-0.20.4^35^. Trimmed reads were aligned to the *Populus trichocarpa* v2.1 transcriptome database^7^ using TopHat2 v2.0.10^36^. A count table was generated using the Python package HTSeq v0.6.1^37^. Python codes for all procedures can be found in document FigShare Python Codes^38^. The raw count table is available under FigShare Raw Count Table^30^.

### Principal component analysis

**Fig. 3:**
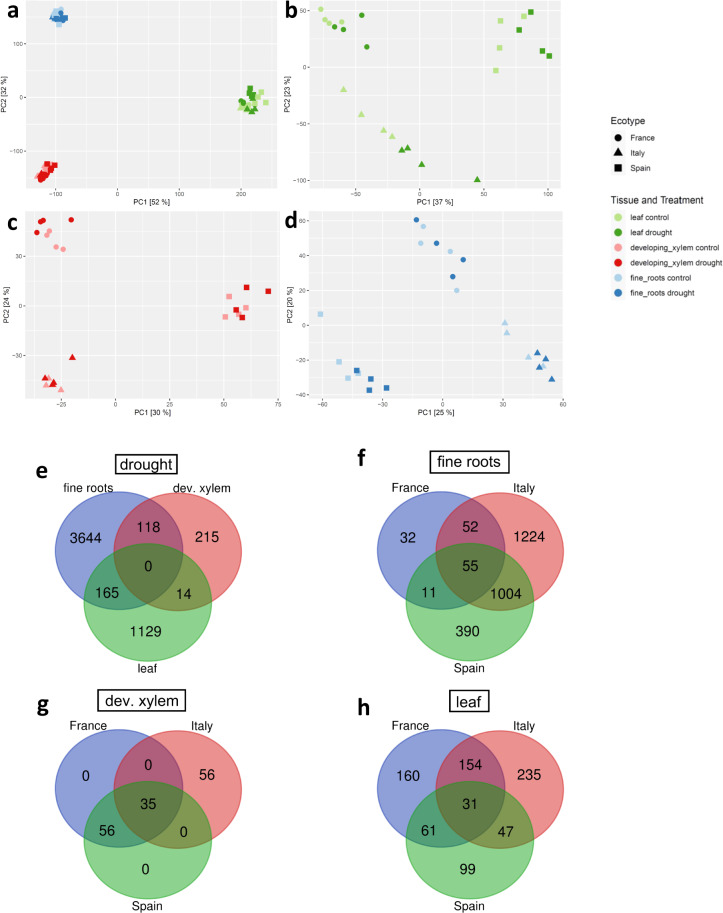
Initial analysis of RNAseq data from control- or drought-stress samples of fine roots, developing xylem and leaf samples of three genotypes of *P. nigra* originating from Spain, France and Italy. (**a**–**d**) Principal component analyses of VST normalized RNA-seq count data (**a**) PCA of all tissues and ecotypes; (**b**) PCA of leaf samples; (**c**) PCA of developing xylem samples; (**d**) PCA of fine root samples; (**e**) Venn diagram showing the distribution of DEGs under drought between the three tissues regardless of the genotype. (**f**–**h**) Venn diagrams showing the intersections of drought-regulated DEGs between the genotypes for fine roots (**f**), developing xylem (**g**) and leaf (**h**).

As a first step of exploratory data analysis, a PCA across all samples of the RNA-seq data was performed. Normalization of counts was performed using the VST (variance stabilizing transformation) function of DESeq2 v1.32.0 package for R^39,40^. The plot of the first two PCs revealed a strong clustering according to tissue type, suggesting strong variation of transcript abundance among tissues (Fig. 3a). Further PCA analyses of leaf (Fig. 3b), developing xylem (Fig. 3c) and fine roots (Fig. 3d) transcriptomes revealed separate clustering of the different genotypes. This result indicates putatively interesting differential gene expression patterns between the different origins (Fig. 3b–d). PCA analysis has been performed with R^40^ using the packages DESeq2 v1.32.0^39^. The R code for the PCA is available under Figshare R Code PCA^41^.

### Initial analysis of differentially expressed genes

Analyses of the count data were done with the R package DESeq2, version 1.34.0^39^ Normalisation of count tables was done based on the ‘median ratio method’^42^ implemented in the function ‘estimateSizeFactors’. We applied an unspecific filtering^43^ to keep only those genes to which at least one read per million reads of library size aligned in at least four samples.

The analysis of differential expression was carried out by fitting two-factorial negative binomial generalized linear models (function ‘DESeq’) to the count data. To assess the significance of a ‘treatment’ main effect, a full model with ´treatment´ and ‘genotype’ main effects was set up, against which a reduced model without ‘treatment’ main effect was tested with the function ‘nbinomLRT’. The sets of genes showing a significant drought main effect were used for a genotype specific analysis of drought effects. For this purpose the data set was split according to tissue and genotype, and for each combination of tissue and genotype, a full model with ‘treatment’ main effect was set up, against which a reduced model with intercept only was tested with the function ‘nbiomLRT’. The R code for the DEG analysis is available under Figshare R Code DEG analysis^44^. Venn diagrams have been made using an online Venn diagram tool (https://bioinformatics.psb.ugent.be/webtools/Venn/).

Analysis of the drought main effect, i.e. without differentiation of the genotypes revealed that the majority of differentially expressed genes (DEGs) were tissue specific under drought (92.8% in fine roots, 62% in the developing xylem and 86.3% in the leaf, Fig. 3e). Interestingly, no shared gene was found to be differentially regulated in all three tissues. When these drought related DEGs shown in Fig. 3e were further analyzed on genotype level, it was found that the majority of drought-related DEGs in fine roots (Fig. 3f) and leaves (Fig. 3h) were genotype specific. However, in the developing xylem (Fig. 3g) there were no genotype specific DEGs found for the Spanish and French genotype. This initial analysis shows, that this dataset can be used to identify conserved drought acclimation processes that are shared by all three genotypes, as well as genotype specific drought responses of *P. nigra*.

## Data Availability

Figshare R Code DEG analysis: 10.6084/m9.figshare.19382594.v1^44^. Figshare Python Codes: 10.6084/m9.figshare.17031869.v2^38^. Figshare R Code for PCA analysis: 10.6084/m9.figshare.17031884.v2^41^.

## Online-only Table

**Online-only Table 1:** RNA sample quality and statistics of sequencing data for each sample in this study. Mapping rate = mapped reads*100/clean reads.

Sample id	genotype	treatment	tissue	raw reads	clean reads	mapped reads	mapping rate (%)	accession	SRP number
pn-gxd001	french	control	dev. xylem	26347794	26219083	24873295	94.9	SRS1884972	SRP095832
pn-gxd002	french	control	dev. xylem	21968470	21834252	20653438	94.6	SRS1884983	SRP095832
pn-gxd003	french	control	dev. xylem	25535311	25432719	24082611	94.7	SRS1884974	SRP095832
pn-gxd004	french	control	dev. xylem	24944154	24788885	23425381	94.5	SRS1884973	SRP095832
pn-gxd005	french	drought	dev. xylem	25869206	25760917	24485336	95.0	SRS1884981	SRP095832
pn-gxd006	french	drought	dev. xylem	23306794	23187681	22037346	95.0	SRS1884979	SRP095832
pn-gxd007	french	drought	dev. xylem	21117296	21016671	19963358	95.0	SRS1884976	SRP095832
pn-gxd008	french	drought	dev. xylem	24135455	24027452	22816016	95.0	SRS1884971	SRP095832
pn-gxd009	italian	control	dev. xylem	25450632	25336566	24023585	94.8	SRS1884984	SRP095832
pn-gxd010	italian	control	dev. xylem	26200813	26077867	24651881	94.5	SRS1884988	SRP095832
pn-gxd011	italian	control	dev. xylem	29039279	28872821	27344418	94.7	SRS1884980	SRP095832
pn-gxd012	italian	control	dev. xylem	29053229	6604984	6272729	95.0	SRS1884989	SRP095832
pn-gxd013	italian	drought	dev. xylem	28736494	28634231	27191223	95.0	SRS1884970	SRP095832
pn-gxd014	italian	drought	dev. xylem	25835119	25727073	24378290	94.8	SRS1884977	SRP095832
pn-gxd015	italian	drought	dev. xylem	28049709	27967115	26538506	94.9	SRS1884987	SRP095832
pn-gxd016	italian	drought	dev. xylem	43867708	43711448	41400778	94.7	SRS1884968	SRP095832
pn-gxd017	spanish	control	dev. xylem	28646361	28525127	26891792	94.3	SRS1884969	SRP095832
pn-gxd018	spanish	control	dev. xylem	21823529	21741207	20586916	94.7	SRS1884975	SRP095832
pn-gxd019	spanish	control	dev. xylem	23440039	23337400	22069986	94.6	SRS1884978	SRP095832
pn-gxd020	spanish	control	dev. xylem	31885434	31742276	30077787	94.8	SRS1884986	SRP095832
pn-gxd021	spanish	drought	dev. xylem	22199898	22103367	20891792	94.5	SRS1884982	SRP095832
pn-gxd022	spanish	drought	dev. xylem	24478986	24372000	23044491	94.6	SRS1884967	SRP095832
pn-gxd023	spanish	drought	dev. xylem	40288279	40147251	37981915	94.6	SRS1884966	SRP095832
pn-gxd024	spanish	drought	dev. xylem	23706143	23583673	22293829	94.5	SRS1884985	SRP095832
pn-gxd025	french	control	fine roots	18475623	18379495	17017509	92.6	SRS2041050	SRP101711
pn-gxd026	french	control	fine roots	26708244	26578726	24589206	92.5	SRS2041049	SRP101711
pn-gxd027	french	drought	fine roots	20093157	20002204	18847673	94.2	SRS2041048	SRP101711
pn-gxd028	french	drought	fine roots	40295194	40123126	37503428	93.5	SRS2041046	SRP101711
pn-gxd029	italian	control	fine roots	20057078	19977533	18660761	93.4	SRS2041047	SRP101711
pn-gxd030	italian	control	fine roots	35300719	18679546	16624171	89.0	SRS2041045	SRP101711
pn-gxd031	italian	drought	fine roots	32071191	31904918	29698037	93.1	SRS2041044	SRP101711
pn-gxd032	italian	drought	fine roots	28132587	28011787	26142944	93.3	SRS2041043	SRP101711
pn-gxd033	spanish	control	fine roots	21947960	21850864	20520694	93.9	SRS2041042	SRP101711
pn-gxd034	spanish	control	fine roots	23484410	23384451	21969301	93.9	SRS2041041	SRP101711
pn-gxd035	spanish	drought	fine roots	24674183	24570824	23027334	93.7	SRS2041040	SRP101711
pn-gxd036	spanish	drought	fine roots	25154915	25061134	23472214	93.7	SRS2041039	SRP101711
pn-gxd037	french	control	fine roots	22974804	22865346	21355856	93.4	SRS2041038	SRP101711
pn-gxd038	french	control	fine roots	26195431	26072182	24281472	93.1	SRS2041037	SRP101711
pn-gxd039	french	drought	fine roots	20610252	20514602	19152381	93.4	SRS2041036	SRP101711
pn-gxd040	french	drought	fine roots	20035140	19931441	18093161	90.8	SRS2041035	SRP101711
pn-gxd041	italian	control	fine roots	28268920	28164055	26344897	93.5	SRS2041034	SRP101711
pn-gxd042	italian	control	fine roots	19010791	18944333	17664174	93.2	SRS2041033	SRP101711
pn-gxd043	italian	drought	fine roots	17776436	17706309	16476970	93.1	SRS2041032	SRP101711
pn-gxd044	italian	drought	fine roots	17781760	17692051	15716453	88.8	SRS2041031	SRP101711
pn-gxd045	spanish	control	fine roots	18083933	17999123	16886475	93.8	SRS2041029	SRP101711
pn-gxd046	spanish	control	fine roots	22195654	22101928	20712694	93.7	SRS2041030	SRP101711
pn-gxd047	spanish	drought	fine roots	27659146	27545602	25615449	93.0	SRS2041028	SRP101711
pn-gxd048	spanish	drought	fine roots	22454164	22340090	20994158	94.0	SRS2041027	SRP101711
pn-gxd049	french	control	leaf	20060742	19948501	18726886	93.9	SRS2041026	SRP101711
pn-gxd050	french	control	leaf	17726230	17618729	16588220	94.2	SRS2041025	SRP101711
pn-gxd051	french	drought	leaf	17135319	17066132	16131181	94.5	SRS2041024	SRP101711
pn-gxd052	french	drought	leaf	27662673	27551365	25997698	94.4	SRS2041023	SRP101711
pn-gxd053	italian	control	leaf	20753033	20664242	19537479	94.5	SRS2041022	SRP101711
pn-gxd054	italian	control	leaf	20742033	20644890	19512057	94.5	SRS2041021	SRP101711
pn-gxd055	italian	drought	leaf	17856205	17775191	16838355	94.7	SRS2041020	SRP101711
pn-gxd056	italian	drought	leaf	18688040	18581365	17589133	94.7	SRS2041019	SRP101711
pn-gxd057	spanish	control	leaf	18482734	18406176	17383881	94.4	SRS2041018	SRP101711
pn-gxd058	spanish	control	leaf	20083897	20006120	18929749	94.6	SRS2041017	SRP101711
pn-gxd059	spanish	drought	leaf	19171934	19095469	18048113	94.5	SRS2041016	SRP101711
pn-gxd060	spanish	drought	leaf	19690261	19617084	18523190	94.4	SRS2041015	SRP101711
pn-gxd061	french	control	leaf	18942527	18836396	17702990	94.0	SRS2041013	SRP101711
pn-gxd062	french	control	leaf	19442585	19330313	18143930	93.9	SRS2041014	SRP101711
pn-gxd063	french	drought	leaf	31703424	31569834	29696014	94.1	SRS2041011	SRP101711
pn-gxd064	french	drought	leaf	19625738	19519758	18289694	93.7	SRS2041012	SRP101711
pn-gxd065	italian	control	leaf	20237232	20135453	19002250	94.4	SRS2041010	SRP101711
pn-gxd066	italian	control	leaf	31513962	31364880	29611859	94.4	SRS2041009	SRP101711
pn-gxd067	italian	drought	leaf	20359403	20254957	19113711	94.4	SRS2041007	SRP101711
pn-gxd068	italian	drought	leaf	23370237	23253368	21970933	94.5	SRS2041008	SRP101711
pn-gxd069	spanish	control	leaf	22973019	22864102	21561281	94.3	SRS2041005	SRP101711
pn-gxd070	spanish	control	leaf	24991988	24866395	23460473	94.3	SRS2041003	SRP101711
pn-gxd071	spanish	drought	leaf	33555678	33431609	31557396	94.4	SRS2041006	SRP101711
pn-gxd072	spanish	drought	leaf	20313704	20233878	19147530	94.6	SRS2041004	SRP101711
